# Supplementary material for: Soluble Transferrin Receptor as Iron Deficiency Biomarker: Impact on Exercise Capacity in Heart Failure Patients
Source: J Pers Med. 2023 Aug 21;13(8):1282. doi: 10.3390/jpm13081282 (PMC10455097; doi:10.3390/jpm13081282)
Supplement: Supplementary file 1 [file jpm-13-01282-s001.zip › jpm-2523872-supplementary.pdf]

**Table S1.** Mean±SD sTfR levels and p-values according to impaired vs preserved submaximal exercise capacity (defined as 6MWT distance <300 meters), and according to impaired vs preserved global QoL (overall summary score of the Minnesota Living with Heart Failure Questionnaire ≥upper tertile)

| | **Submaximal exercise capacity** | | |
|---|---|---|---|
| | **Preserved submaximal exercise capacity (n=111)** | **Impaired exercise capacity (n=91)** | **P-value** |
| sTfR levels, mean (SD) | 1.32 (0.61) | 1.53 (0.72) | p=0.030 |
| | **Quality of Life** | | |
| | **Preserved QoL (n=137)** | **Impaired QoL (n=69)** | **P-value** |
| **Overall score** | | | |
| sTfR levels, mean (SD) | 1.38 (0.61) | 1.53 (0.77) | p=0.151 |
| **Physical dimension** | | | |
| sTfR levels, mean (SD) | 1.43 (0.74) | 1.44 (0.42) | p=0.846 |
| **Emotional dimension** | | | |
| sTfR levels, mean (SD) | 1.37 (0.61) | 1.55 (0.78) | p=0.058 |
| **Social dimension** | | | |
| sTfR levels, mean (SD) | 1.37 (0.60) | 1.55 (0.79) | p=0.067 |

**Table S2.** Adjusted GAM models to explore the parametric and non-parametric associations between sTfR and submaximal exercise capacity (distance walked in the 6MWT in meters) and QoL scores.

| | **Log (sTfR)** | |
|---|---|---|
| **Dependent variables** | **Parametric p-value** | **Non-parametric p-value** |
| **Submaximal exercise capacity** | | |
| Distance walked in the 6 minutes walking test (meters) | <0.001 | 0.138 |
| **Quality of Life measured with the Minnesota Living with HF Questionnaire** | | |
| Overall summary score (Global QoL) | 0.006 | 0.615 |
| Physical dimension | 0.002 | 0.398 |
| Emotional dimension | 0.061 | 0.596 |
| Social dimension | 0.418 | 0.548 |

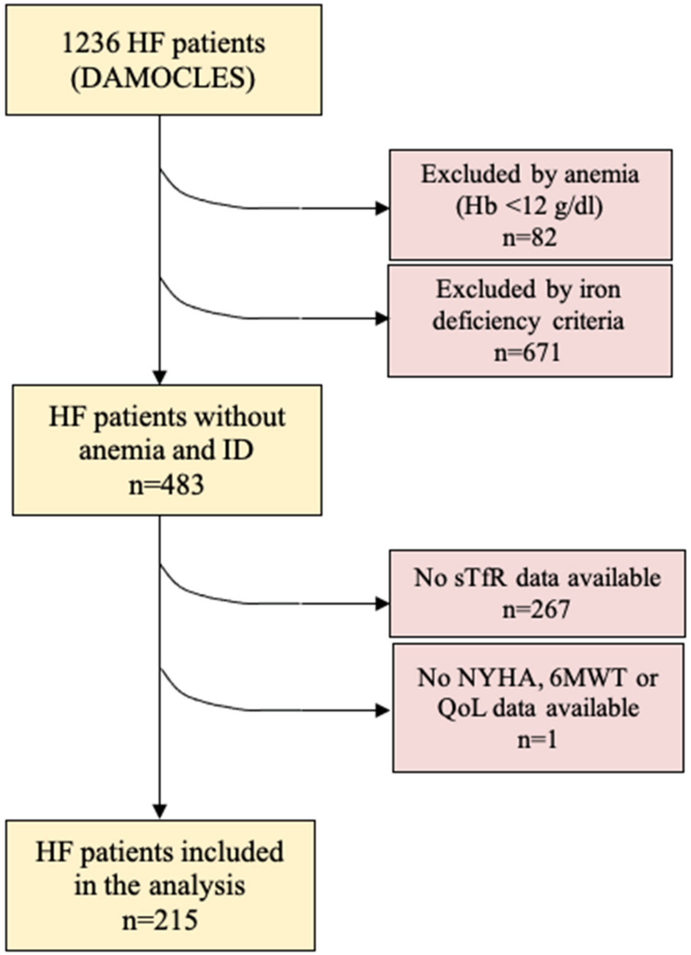


**Figure S1.** Patient flow diagram of the enrolment in the DAMOCLES study for present analysis.

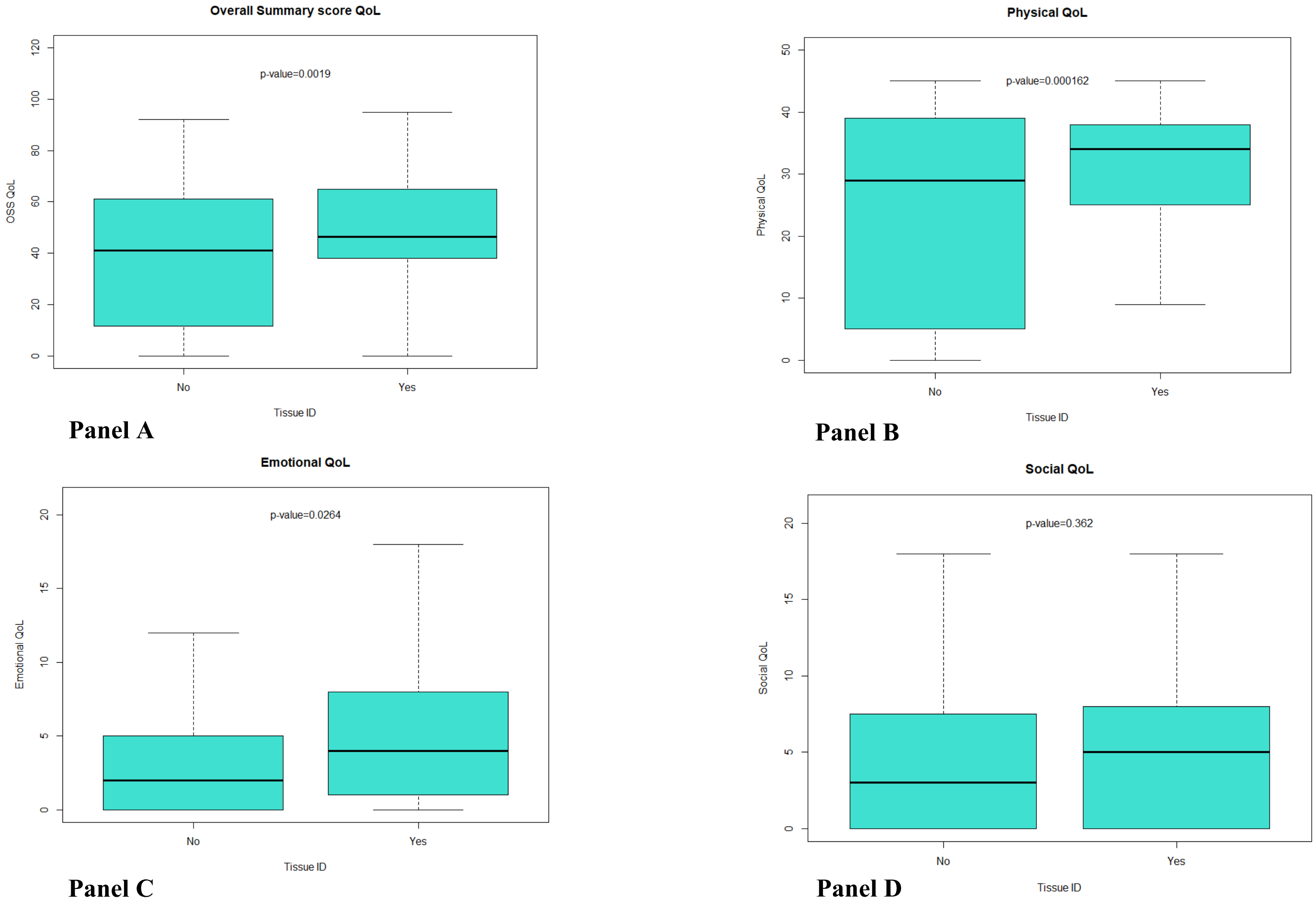
Overall Summary score QoL
p-value=0.0019
OSS QoL
0
20
40
60
80
100
120
No
Yes
Tissue ID
Panel A
Physical QoL
p-value=0.000162
Physical QoL
0
10
20
30
40
50
No
Yes
Tissue ID
Panel B
Emotional QoL
p-value=0.0264
Emotional QoL
0
5
10
15
20
No
Yes
Tissue ID
Panel C
Social QoL
p-value=0.362
Social QoL
0
5
10
15
20
No
Yes
Tissue ID
Panel D

**Figure S2. Panel A.** Boxplots showing the mean and standard deviation of the overall summary score (**Panel A**) and the physical (**Panel B**), emotional (**Panel C**) and social (**Panel D**) dimension scores of the Minnesota Living with Heart Failure Questionnaire (higher scores meaning worse global QoL) according to the presence of tissue ID (Tissue ID [+]) or absence (Tissue ID [-]).

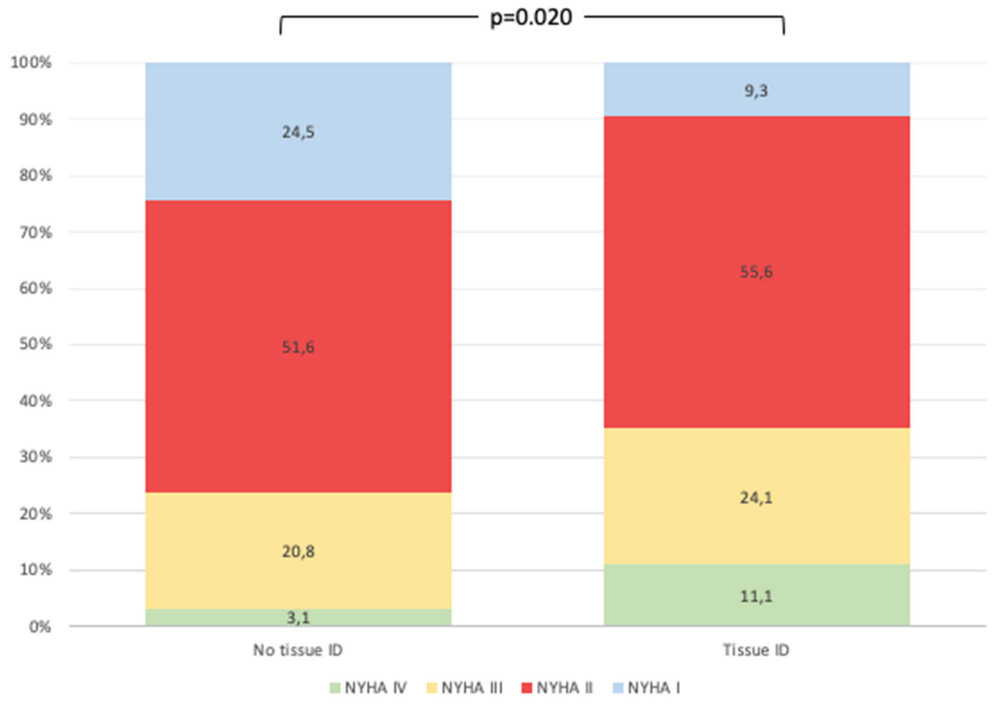


**Figure S3.** Distribution of patients according to NYHA and impairment in submaximal exercise capacity (defined as 6MWT distance<300 meters) according to tissue ID status.
